# Comparative analysis of length-weight relationships and condition factors of two congeneric rockcod species from the shores of King George Island, Antarctica

**DOI:** 10.7717/peerj.20513

**Published:** 2026-01-08

**Authors:** Seungyeon Lee, Mi-Hyun Park, Sachithra Amarin Hettiarachchi, Jihun Kim, Yong-Woo Lee, Jin-Hyoung Kim

**Affiliations:** 1 Division of Life Sciences, Korea Polar Research Institute, Incheon, Republic of South Korea; 2 Polar Science, University of Science and Technology, Incheon, Republic of South Korea; 3 Department of Zoology, University of British Columbia, Vancouver, British Columbia, Canada; 4 Office of Science and Technology, National Marine Fisheries Service, National Oceanic and Atmospheric Administration, Silver Spring, MD, United States of America

**Keywords:** Length-weight relationships, Condition factor, Antarctic fish, *Notothenia coriiceps*, *Notothenia rossii*, Notothenioids, Antarctica

## Abstract

Length-weight relationships (LWR) and Fulton’s condition factors (*K*) of two notothenioid species, *Notothenia rossii* and *Notothenia coriiceps*, were assessed using 295 and 148 specimens, respectively. Fish samples were collected from two different locations: the wharf of the King Sejong Station and Chotdaebawi in Maxwell Bay, King George Island, from January to February 2023. The coefficient of determination (*R*^2^) of linear regressions on LWR ranged from 0.935 to 0.970, and the *b* values ranged from 3.05 to 3.27. Only *N. coriiceps* caught in the wharf exhibited positive allometric growth, while *N. coriiceps* from Chotdaebawi and *N. rossii* from both locations demonstrated isometric growth. The estimated condition factors (*K*) indicated that all groups of *N. rossii* and *N. coriiceps* exhibited favorable growth conditions in their respective biotopes. These findings enhance our understanding of the similarities and differences in fundamental biological characteristics, ecological aspects, and growth conditions of two important congeneric Antarctic fish species.

## Introduction

At the highest latitudes, the endemic perciform suborder Notothenioidei constitutes 77% of species diversity, 92% of the abundance, and 91% of biomass in the Antarctic region (Eastman, 2005; La Mesa, Eastman & Vacchi, 2004). Notothenioidei plays a crucial role in the Antarctic food web, particularly at the mid-trophic levels (Barrera-Oro, 2002; Eastman, 1985; Stefanov, 2022). Among the notothenioids, the marbled rockcod, *Notothenia rossii* Richardson 1844, and the black rockcod, *Notothenia coriiceps* Richardson 1844, inhabit demersal zones in coastal areas (Eastman, Barrera-Oro & Moreira, 2011; Tiedtke & Kock, 1989). *Notothenia rossii* undergoes a pronounced ontogenetic habitat shift. Juveniles inhabit shallow inshore zones associated with macroalgal or kelp beds, typically at depths less than 100 m, for approximately 5 to 7 years (Barrera-Oro, La Mesa & Moreira, 2014; Burchett, 1982; Hollyman et al., 2021; Uzunova, Kenderov & Stefanov, 2021). Upon reaching sexual maturity, individuals adopt a more semi-pelagic behavior and migrate to deeper offshore shelf areas, generally ranging from 100 to 350 m, where adult populations are distributed (Burchett, 1982; Hollyman et al., 2021). In comparison, *N. coriiceps* is a bottom-feeding predator in benthic coastal zones (Gon & Heemstra, 1990; Uzunova, Kenderov & Stefanov, 2021). It also exhibits a broad bathymetric range, from surface waters to depths of 550 m conditions, and its early pelagic stages are widely distributed across the Antarctic, indicating circum-Antarctic dispersal (Gon & Heemstra, 1990; Palm et al., 1998).

These distinct habitat preferences and life history strategies highlight the importance of assessing their physiological condition under varying environmental conditions. Such ecological differences suggest that growth patterns and physiological conditions may vary under different environmental conditions. Numerous studies have used statistical length-weight relationship (LWR) and condition factors to assess the overall physiological well-being of populations across different seasons and ecological conditions (Birecikligil et al., 2016; Froese, 2006; Le Cren, 1951; Ouahb et al., 2021; Pouladi et al., 2020; Stergiou & Moutopoulos, 2001).

The LWR for a fish population is a valuable tool for estimating a fish’s weight based on its length, as length is generally easier to measure accurately in the field than weight. It also provides insights into the well-being and body condition of fish (Froese, Thorson & Reyes Jr, 2014) and informs future studies on fish populations and stock assessments (Mehanna & Farouk, 2021). LWRs in fishes vary due to various factors such as seasonal variations, gonad maturity, sex differences, dietary preferences, stomach contents, health, environmental stresses, food accessibility, and habitat characteristics (Bagenal & Tesch, 1978; Pauly, 1983; Sungur et al., 2022).

Complementing LWR, Fulton’s condition factor (*K*) assesses the general well-being, fatness, or condition of fish, based on the premise that individuals with higher weights for a given length are in better condition (Froese, 2006; Ricker, 1968; Tsoumani et al., 2006). *K* is calculated from length and weight, but it is influenced by a range of environmental and biological factors. As such, *K* can be used to compare the relative health of fish populations living under different feeding densities, climates, and habitat conditions (Weatherley, Gill & Casselman, 1987). However, while *K* provides a useful indicator of population-level health in the context of environmental factors, additional variation often arises due to more specific biological influences. According to Ricker (1975), *K* may vary among populations of the same species or even within a single population across different years, depending on the nutritional status and activity. At the interspecific level, Ragheb (2023) reported that differences in body shape are likely responsible for variation in mean *K* between species. Thus, *K* serves as a useful proxy for understanding how environmental and biological factors interact to influence fish condition across populations or species.

Building on this foundation, the present study aims to: (1) estimate the parameters of the LWR and *K* for two ecologically important Antarctic notothenioid species (*N. rossii* and *N. coriiceps*) based on samples from two locations on King George Island in the Antarctic Peninsula; and (2) compare these growth characteristics between species and sampling localities. Given the environmental and logistical challenges of conducting fish sampling in the Antarctic Peninsula, our findings provide valuable information and insights into the biological and ecological characteristics of these populations.

## Materials & Methods

### Collection of fish samples

**Figure 1:**
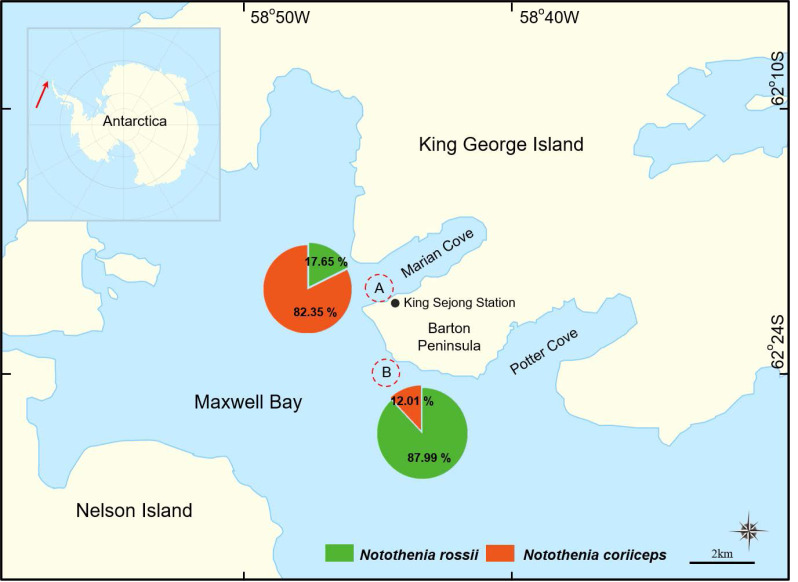
A map of two sampling locations in Maxwell Bay, Antarctica. (A) The wharf of the King Sejong Station (62°13′19.0″S 58°47′13.6″W), (B) Chotdaebawi (62°14′18.7″S 58°46′36.9″W). The catch composition of each sampling location is visually represented through pie charts, delineating the percentage by the number of fish, with each fish figure denoting the predominant species in its respective location.

Fish samples were collected using hook-and-line and fish traps from January to February 2023 at two sampling locations around King Sejong Station in Maxwell Bay, King George Island, Antarctica: the wharf of the King Sejong Station (hereafter Location A, 62°13′19.0″S 58°47′13.6″W) and Chotdaebawi (hereafter Location B, 62°14′18.7″S 58°46′36.9″W), Maxwell Bay, King George Island, Antarctica (Fig. 1). Both sampling locations were near the shore, approximately three km apart along the coast. Depth ranged from 2 to 30 m, depending on the distance from the shore, with the site at the wharf (Location A) being shallower than the one at Chotdaebawi (Location B). The fish trap used in this study was a cylindrical funnel trap, measuring 60 cm in length and 30 cm in diameter. A total of five traps were deployed exclusively at Location A during the first sampling period. These traps were deployed on the bottom, with heavy sinkers placed inside to ensure they settled securely on the seafloor. The traps were set at depths ranging from 0.5 m to 3 m, depending on tidal fluctuations. However, their catch efficiency was low, yielding only approximately 20 fish over a one-week period. Due to this low efficiency, the fish traps were not used in subsequent sampling periods. The hook-and-line sampling method was used at both sampling locations. At Location A, two hook-and-line setups were used for two weeks, with fishing sessions lasting 1–2 h per day. At Location B, eight hook-and-line setups were deployed over three days, with fishing sessions lasting 2–3 h per day. Due to differences in sampling conditions and intensity between the locations, and because the main focus of the study was growth metrics rather than abundance, catch abundance was not standardized by unit effort for direct comparisons.

A total of 443 individuals of two species, *N. rossii* (*n* = 295) and *N. coriiceps* (*n* = 148), were sampled (Table 1). Upon capture, the total length (TL) to the nearest 1 mm and total weight (W) to the nearest 1 g of each individual were immediately measured using a fish length measuring board and an electronic scale. The methods employed in this study adhered to the ethical guidelines of the Institutional Ethics Committee of Korea Polar Research Institute (KOPRI). The animal study protocol was approved by the Institutional Ethics Committee of KOPRI (KACC2301_012).

**Table 1:** Total length and weight data of two Antarctic notothenioid species captured in the coastal waters near King Sejong Station from January to February 2023. Sampling location A: the wharf of the King Sejong Station, Sampling location B: Chotdaebawi. The asterisk denotes statistically significant difference (*p* < 0.01) between groups, based on *post hoc* multiple comparison tests.

Species	*n*	Sampling location	Total length (mm)			Total weight (g)		
			Mean	Min	Max	Mean	Min	Max
*N. rossii*	24	A	274.0	170	350	318.94	60.37	742.7
	271	B	312.2*	185	470	432.50	71.54	1,229.2
*N. coriiceps*	111	A	273.7	180	420	381.32	88.76	1,336.55
	37	B	282.7	220	410	345.27	135.13	962.37

### Length-weight relationship

LWR was estimated for each species per sampling location using the formula *W* = *aL*^*b*^ (Le Cren, 1951). Length is a measure in one dimension, whereas weight, influenced by volume, exists in three dimensions. The parameters *a* and *b* were estimated through linear regressions with the Analysis ToolPak in Microsoft Excel 2021 (Sangun, Akamca & Akar, 2007), after applying the common logarithm transformations to both sides of the equation: log *W* = log *a* + *b* log *L* (Zar, 1984). The *b* value can vary among fish populations from different locations, genders, or life stages (larval, juveniles, and adults), but remains consistent within a given group (Ricker, 1973). Additionally, we examined species-specific growth patterns (allometric *vs.* isometric) for each location based on the *b* value (see Statistical analysis section for details).

### Fulton’s condition factor

Fulton’s condition factor (*K*) was calculated based on the formula: *K* = 100 × *W*/*L*^3^ (Ricker, 1975). A fish with a *K* value higher than 1 indicates that the fish is in good growth condition, implying overall good well-being.

### Statistical analysis

The statistical significance of the estimated *a* and *b* values of the LWR were evaluated based on the 95% confidence intervals (CIs) from the regression outputs. The strength of the relationship between variables was evaluated based on the coefficient of determination (*R*^2^). To evaluate whether the estimated *b* values differed statistically (*p* < 0.05) from the ideal isometric growth value (*b* = 3), *t*-tests were used. A statistically significant difference between the estimated mean *b* value and 3 (*p* < 0.05), based on a *t*-test, indicates either positive allometric growth (*b* > 3, A^+^) or negative allometric growth (*b* < 3, A^−^) of a fish population. Conversely, if the *b* value is not statistically different from 3 (*p* > 0.05), it suggests isometric growth (*b* = 3, A^0^) of a fish population.

Statistical comparisons of fish lengths, weights, and condition factors were performed using a two-way ANOVA including interaction term with the *car* package in R (version 4.5.0). When a significant effect was detected in the ANOVA model (*p* < 0.05), Tukey-adjusted pairwise comparisons were conducted using the *emmeans* package to test for differences between the groups.

## Results

In this study, significant differences in catch proportions, size, and growth patterns were observed between the two congeners, *N. rossii* and *N. coriiceps*, collected from two distinct sampling locations. The catch proportions differed markedly by sampling location: 17.65% *N. rossii* and 82.35% *N. coriiceps* in location A, and 87.99% *N. rossii* and 12.01% *N. coriiceps* in location B (Fig. 1).

**Figure 2:**
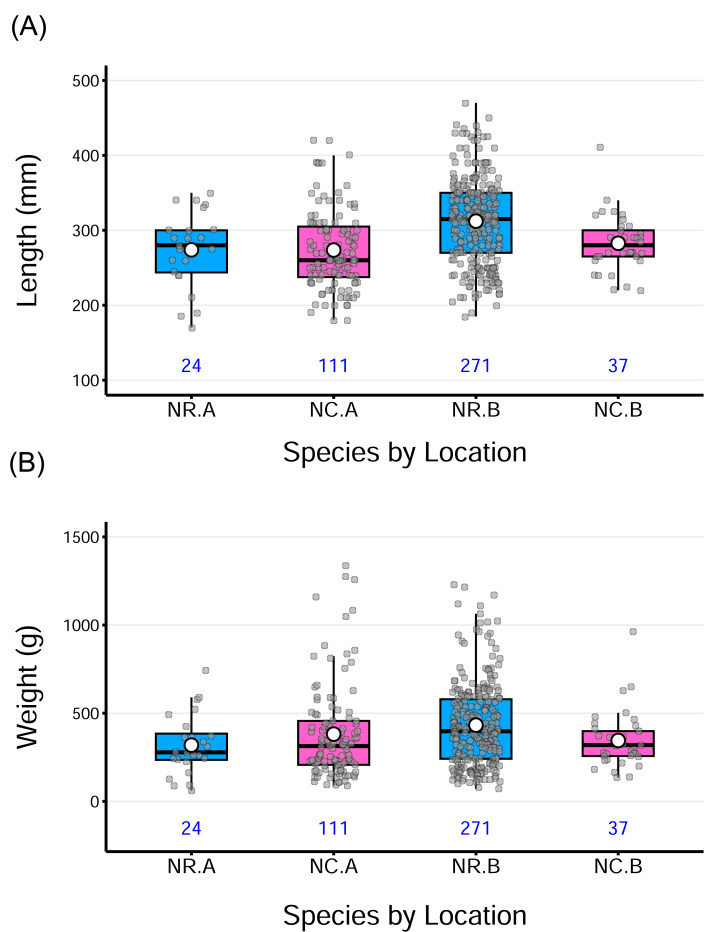
Distribution of total fish lengths and weights by species from two sampling locations. The horizontal line within each box indicates the median, while the solid white circle represents the sample mean for each group. Gray points show individual observations, jittered horizontally to reduce overlap. The numbers under each box denote the sample size for each group. Acronyms for the group labels on the *X*-axis as follows: NR = *Notothenia rossii*, NC = *Notothenia coriiceps*; A = the wharf of King Sejong Station, B = Chotdaebawi.

The samples of both *N. rossii* and *N. coriiceps* encompassed a wide size range, covering size classes from juveniles through to adult stages (Table 2). The boxplots further illustrate these patterns, showing substantial variation in both lengths and weights, with a few outliers at both ends of the distributions in some groups. Despite differences in sample size, the distributions appear generally balanced, as reflected by the close proximity of medians and means. A slight right skewness was observed in certain groups, where the mean exceeded the median (Fig. 2).

**Table 2:** Length-weight relationships and condition factor of two sister Antarctic fishes. Positive allometric growth (A^+^) is indicated by a significant difference between the *b* value and 3 (*p* < 0.05), while isometric growth (A^0^) is observed when the *b* value is not statistically different from 3.

Species	Sampling location	*n*	LWR regression parameters						Condition factor
			*a*	95% CI of *a*	*b*	95% CI of *b*	Growth pattern	*R* ^2^	*K*
*N. rossii*	A	24	7.75 × 10^−6^	1.23 × 10^−6^–4.8 9 × 10^−5^	3.10	2.78–3.43	A^0^	0.946	1.40 ± 0.04
	B	271	9.76 × 10^−6^	5.88 × 10^−6^–1.62 × 10^−5^	3.05	2.96–3.14	A^0^	0.965	1.30 ± 0.01
	Total	295	1.02 × 10^−5^	6.89 × 10^−6^–1.51 × 10^−5^	3.04	2.97–3.11	A^0^	0.945	1.31 ± 0.01
*N. coriiceps*	A	111	3.45 × 10^−6^	1.88 × 10^−6^–6.32 × 10^−6^	3.27	3.17–3.38	A^+^	0.970	1.61 ± 0.02
	B	37	5.54 × 10^−6^	1.10 × 10^−6^–2.78 × 10^−5^	3.17	2.88–3.46	A^0^	0.935	1.44 ± 0.03
	Total	148	4.48 × 10^−6^	2.39 × 10^−6^–8.39 × 10^−6^	3.22	3.11–3.33	A^+^	0.958	1.57 ± 0.02

**Notes.**

CI Confidence interval

A two-way ANOVA on length revealed a significant main effect of location on length, *F*_(1,439)_ = 9.24, *p* = 0.0025, indicating that fish from location B were longer, on average, than those from location A, across both species. The main effect of species, was not significant, suggesting no overall difference in length between the two species. Similarly, the species-by-location interaction was not statistically significant for length (*p* = 0.061), though marginal, suggesting a potential difference in how location affects length across species. Post hoc pairwise comparisons (Tukey-adjusted) indicated that *N. rossii* from location B was significantly longer than the other groups, possibly contributing to the observed main effect of location to be significant. Despite these findings, the overall model fit was very low (adjusted *R^2^* = 9.3%), suggesting that length variation is mostly driven by individual differences rather than species or location. Boxplots (Fig. 2A) visually support these results, showing modest group-level differences with substantial overlap. Detailed analysis of variance (ANOVA) results for length, as well as weight and fish condition, are provided in the supplementary information.

For weight, a two-way ANOVA revealed a significant interaction between species and location, *F*_(1,439)_ = 4.89, *p* = 0.028, indicating that the effect of one factor depends on the level of the other. However, the main effect of species and location was not significant, and the model accounted for only a small proportion of variance in weight (adjusted *R^2^* = 1.61%). Tukey-adjusted pairwise comparisons indicated that no specific group differences were statistically significant (*p* > 0.05). While the interaction term was statistically significant, the absence of significant main effects and pairwise comparisons, combined with the small effect size and low model fit, suggests that the observed interaction is subtle and not practically meaningful in terms of distinct group-level differences. The overall pattern appears to be driven by variability within groups rather than by clear differences between them. This pattern is also visible in the distribution of weight data across groups (Fig. 2B).

### Length-weight relationship and growth pattern

**Figure 3:**
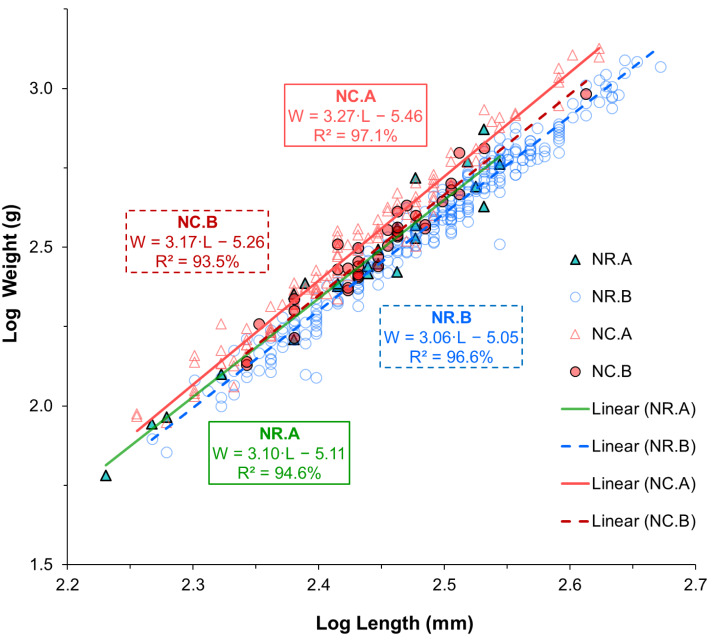
Linear regression lines fitted to log-transformed length and weight data of two Antarctic fish species from two different locations. Triangle symbols represent fish from sampling location A, while circle symbols represent fish from sampling location B. Solid lines indicate the regression fits for samples from location A, while dotted lines represent those from location B. Different color schemes are used to differentiate species-location groups: green for *N. rossii* (NR) from location A, blue for NR from location B, pink for *N. coriiceps* (NC) from location A, and red for NC from location B.

The LWR, the coefficient of determination (*R*^2^), the 95% confidence interval (CI) for *a* and *b* estimates, the growth pattern, and *K* value for the two species are shown in Table 2. The LWR regression lines and individual observations are shown on a log–log scale for each species-location group in Fig. 3. According to the statistical results, the correlations between length and weight for all groups of each species and sampling location were strong, with *R^2^* values greater than 0.9 for all cases. The coefficient of determination (*R*^2^) was significant for all groups of each species and sampling location (*p* < 0.001). The estimates of parameter *b* for all groups ranged between 3.05 and 3.27. According to the *b* parameter estimates, *N. rossii* exhibited isometric growth (A_0_), with *b* values of 3.10 at location A, 3.05 at location B, and 3.04 when data from both locations were combined (Table 2). *Notothenia coriiceps* showed significant positive allometric growth (A^+^), with *b* values of 3.27 (95% CI [3.17–3.38]) at location A and 3.22 in the combined sample (*t-* tests, *p* < 0.001). However, the sample from location B exhibited isometric growth (A_0_), with a *b* value of 3.17 and a 95% CI of 2.88–3.46.

### Fulton’s condition factor (*K*)

**Figure 4:**
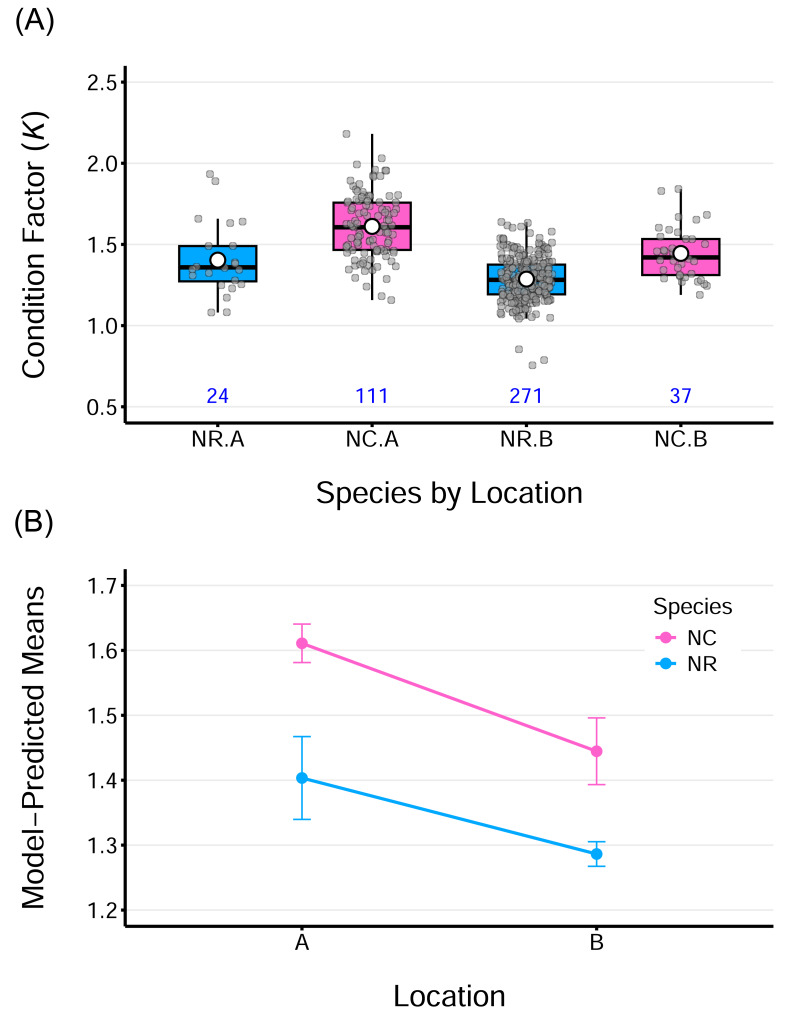
Condition factor (*K*) for two species sampled at two locations. (A) Boxplots of Fulton’s condition factor (*K*) for two species, *N. rossii* (NR) and *N. coriiceps* (NC), sampled at two locations: A (the wharf of King Sejong Station) and B (Chotdaebawi). Numbers below each box indicate the sample size for each group. (B) Line plot of ANOVA model–predicted mean condition factors by species from the two sampling locations. Each line connects species-specific predicted means. The condition factor of NC species is consistently higher, regardless of location. Fish from location A consistently show higher mean condition than those from location B, irrespective of species. Parallel lines suggest an additive effect of location, consistent with the insignificant interaction term in the model. Vertical bars represent 95% confidence intervals.

The average *K* values ranged from 1.30–1.40 for *N. rossii* and 1.44–1.61 for *N. coriiceps* (Table 2), were above 1 for both species regardless of sampling locations. This indicates that both species are in a state of good body condition, characterized by fat accumulation. Boxplots of fish condition show that the data are relatively symmetric and evenly distributed across groups, with minimal outliers (Fig. 4A). A two-way ANOVA on fish conditions revealed significant main effects of species, *F*_(1,439)_ = 64.96, *p* < 0.0001, and location, *F*_(1,439)_ = 39.00, *p* < 0.0001. However, the interaction between species and location was not significant, *F*_(1,439)_ = 1.18, *p* = 0.279, indicating that the effects of species and location on condition were additive. This additive effect of location is more evident in the plot of model-predicted means by group, which highlights consistently higher condition values for fish from location A compared to those from location B, for both species (Fig. 4B). The ANOVA model for fish conditions explained approximately 43% of the overall variance in the data (adjusted *R^2^* = 42.8%). Considering the simplicity of the model, with only two categorical factors, each having two levels, this indicates a relatively strong model fit and suggests that both factors had measurable effects.

## Discussion

This study identified distinct interspecific differences, potentially influenced by location, in catch proportions, body size (length and weight), LWRs, and growth conditions between two sympatric notothenioid species, *N. rossii* and *N. coriiceps*, collected from two nearby nearshore sites. While both species generally exhibited good body condition, their patterns of variation between locations differed, suggesting that species-specific ecological or physiological traits may influence how they respond to environmental factors such as water depth, prey availability, and habitat structure. The observed spatial variation in growth and size likely reflects the combined effects of these species-specific traits and local habitat variation. These findings highlight the importance of considering both fine-scale environmental differences and species-specific characteristics when studying local population dynamics.

Catch proportions showed the most notable differences between the two sampling locations. Although precise environmental data (*e.g.*, depth profiles and substrate composition) were not collected, the observed patterns are consistent with known ecological preferences and may reflect variation in depth and bottom conditions. Location A, being shallower, had a higher proportion of *N. coriiceps* (82.35%), consistent with its preference for benthic habitats, particularly those rich in benthic organisms like limpets (Trokhymets et al., 2022; Zamzow et al., 2011). In contrast, *N. rossii* was dominant at the deeper location B (87.99%), consistent with its benthopelagic nature. While the limited sample size may have influenced this result, the close proximity of the two sites (∼3 km) and the concurrent timing of sampling support the interpretation that the observed difference is likely an actual ecological pattern rather than a sampling artifact. The observed difference in catch proportions may also reflect ecological distinctions between the two species, particularly in their habitat preferences. *Notothenia coriiceps* is typically associated with shallower, benthic environments, whereas *N. rossii* has been reported to occupy a broader range of depths and habitats, though still primarily within the benthic demersal zone (Barrera-Oro, 2002; Hollyman et al., 2021). Although our study did not directly assess feeding habits or habitat use, these established ecological traits offer a plausible explanation for the depth-related distribution patterns observed. Our findings are consistent with previous reports of species-specific habitat use and contribute to a better understanding of their spatial distribution in the studied area.

Although substantial variation was observed in body length among individual fish, the statistical analysis suggests a consistent pattern, indicating that environmental conditions at location B may have been more favorable for the growth of *N. rossii*. As it matures, this species undergoes a shift in diet toward energy-rich prey such as krill and fish, which may support its higher growth potential and larger body size. In contrast, *N. coriiceps* feeds on a variety of prey, including amphipods, gastropods (*e.g.*, limpets), algae, and fish, with a notable reliance on benthic invertebrates (Barrera-Oro, 2002; Kock, 1992; Trokhymets et al., 2022). One possible explanation is that environmental factors at location B, such as greater prey availability or reduced competition, may have supported faster growth or contributed to the presence of larger individuals of *N. rossii*. While the current study lacks data to directly test these mechanisms, our findings provide a foundation for developing working hypotheses.

The isometric growth pattern of *N. rossii*, observed in this study, is consistent with findings from Livingston Island (Stefanov, 2022), but differs from studies in other locations, such as King George Island, South Shetland, South Orkney Islands, and Elephant Island, where positive allometric growth has been reported (Balguerías et al., 1989; Eastman, Barrera-Oro & Moreira, 2011; Eastman & Sidell, 2002; Kim et al., 2023; Kock, 1986; Kock & Jones, 2000; Park et al., 2017). *Notothenia coriiceps* exhibited positive allometric growth, with an average *b* value of 3.22 across both locations, consistent with studies from the South Shetland Islands (Eastman, Barrera-Oro & Moreira, 2011; Eastman & Sidell, 2002) and King George Island (Park et al., 2017). However, this contrasts with isometric growth at King George Island (Kim et al., 2023) and negative allometric growth at Livingston Island (Stefanov, 2022). These results, along with previous studies, indicate that growth patterns can vary even within a given species when exposed to different environmental conditions.

Condition factors (*K*) were relatively high for both species, with nearly all sampled individuals having values above 1, except for a few *N. rossii* from location B. While within- and between-group differences in length and weight were not particularly distinct, patterns in *K* were much clearer, as reflected in both the plots and ANOVA results, which showed a relatively strong model fit. Interestingly, condition patterns were opposite to those for length and weight. *Notothenia rossii* at location B had greater observed length and weight, but it had the lowest condition factor. Furthermore, while ANOVA results for length showed significantly greater values at location B on average across species, condition factors were significantly higher at location A for both species. These findings suggest that *N. coriiceps* maintained a higher growth condition than *N. rossii* at both locations, and that increases in length and weight are not necessarily correlated with better growth condition. The insignificant interaction term indicates that the effects of species and location on growth condition are independent and additive.

Differences in *K* between the species are likely related to interspecific morphological traits, with *N. rossii* having a more streamlined body compared to the bulkier *N. coriiceps*, which features a large, wide, and flattened head. Ecologically, *N. coriiceps* is a demersal and sedentary fish, whereas *N. rossii* is benthic and semi-pelagic, characterized by a streamlined, laterally compressed body and narrower head (Barrera-Oro et al., 2019; Casaux, Mazzotta & Barrera-Oro, 1990; Eastman, Barrera-Oro & Moreira, 2011). These morphological and ecological differences may serve as baseline explanations for the variations in length-weight relationships and thus the condition factors observed between the species.

Beyond these considerations, maturity composition and reproductive status during the sampling period may also have influenced the observed *K* values. For *N. rossii*, the samples included both juveniles and adults, and the presence of immature juveniles may have contributed to its lower mean *K* relative to *N. coriiceps*. In contrast, our sampling of *N. coriiceps* (January–February) coincides with the pre-spawning or early spawning season of *N. coriiceps* (Hureau, 1970; Casaux, Mazzotta & Barrera-Oro, 1990; Ferreira et al., 2017). Active feeding and energy accumulation during this phase may have contributed to the elevated *K* values. However, the length data indicate that immature specimens were also present in samples, suggesting that reproductive status alone does not fully explain the patterns in *K*. Based on these observations, the differences in *K* between the two species reflect the species-specific traits shaped by a combination of morphology, reproductive status, maturity composition, and broader life-history characteristics.

Spatially, higher *K* values were observed at location A in both species, which may be linked to enhanced physiological performance associated with benthic habitats potentially offering a richer, higher-energy prey field. However, further research is needed to more accurately identify the intrinsic and extrinsic factors contributing to the observed differences in size distributions and growth conditions. These differences in growth could have important implications for differential survival potential under changing environmental conditions. Higher body condition may enhance resilience to stressors by providing greater energy reserves, while faster growth in size could confer advantages in competition and reproduction.

We consider the implications of our findings are important for the conservation and management of these species in Antarctic waters, as well as for developing working hypotheses for future studies. Understanding their differential growth responses and habitat preferences provides valuable insight for predicting population compositions and dynamics in response to environmental shifts, particularly in polar regions, where impacts of global warming are more pronounced.

## Conclusions

This study provides valuable insights into the ecological and biological differences between two Antarctic notothenioid species, *N. rossii* and *N. coriiceps*, collected from nearby but ecologically distinct locations. Our findings reveal species-specific differences in catch proportions, size distributions, growth patterns, and condition factors, could be shaped in part by habitat characteristics such as depth and prey availability. *Notothenia coriiceps* exhibited positive allometric growth at the shallower location, likely reflecting adaptation to benthic environments with more abundant resources, while *N. rossii* displayed consistent isometric growth across locations, suggesting greater ecological flexibility. Condition factor analysis indicated that increases in size do not always correspond to improved physiological condition, with *N. coriiceps* maintaining higher condition levels despite sometimes having smaller body sizes. These patterns underscore the role of habitat in shaping growth dynamics and suggest that species-specific traits and location independently affect physiological performance. Future research should broaden the geographic scope and incorporate genetic analyses to better understand stock structure, reproductive mixing, differential growth and survivability across habitats, and species-specific responses to environmental change, particularly in the context of global warming.

##  Supplemental Information

10.7717/peerj.20513/supp-1 Supplemental Information 1 Raw data of two Antarctic fish metrics

## Additional Information and Declarations

### Data Availability

The following information was supplied regarding data availability: The raw data is available in the Supplemental File.
